# Quantification of atrial cardiomyopathy disease severity by electroanatomic voltage mapping and cardiac magnetic resonance imaging

**DOI:** 10.1111/jce.16462

**Published:** 2024-12-30

**Authors:** Iain Sim, Jose Alonso Solis Lemus, Christopher O'Shea, Orod Razeghi, John Whitaker, Rahul Mukherjee, Daniel O'Hare, Noel Fitzpatrick, James Harrison, Ali Gharaviri, Louisa O'Neill, Irum Kotadia, Caroline H. Roney, Neil Grubb, David E. Newby, Marc R. Dweck, Pier‐Giorgio Masci, Matthew Wright, Amedeo Chiribiri, Steven Niederer, Mark O'Neill, Steven E. Williams

**Affiliations:** ^1^ Division of Imaging Sciences and Biomedical Engineering King's College London London UK; ^2^ Department of Cardiovascular Scienes University of Birmingham Birmingham UK; ^3^ Department of Cardiology Guy's and St Thomas' NHS Foundation Trust London UK; ^4^ Centre for Cardiovascular Science The University of Edinburgh Edinburgh UK

**Keywords:** atrial fibrillation, bipolar voltage, fibrosis, late gadolinium enhancement

## Abstract

**Introduction:**

Atrial late gadolinium enhancement (Atrial‐LGE) and electroanatomic voltage mapping (Atrial‐EAVM) quantify the anatomical and functional extent of atrial cardiomyopathy. We aimed to explore the relationships between, and outcomes from, these modalities in patients with atrial fibrillation undergoing ablation.

**Methods:**

Patients undergoing first‐time ablation had disease severities quantified using both Atrial‐LGE and Atrial‐EAVM. Correlations between modalities and their relationships with clinical features and arrhythmia recurrence were assessed.

**Results:**

In 123 atrial fibrillation patients (60 ± 10 years), Atrial‐EAVM was moderately correlated with Atrial‐LGE (*r* = .34, *p* < .001), with a mean fibrosis burden of 47.2% ± 14.91%. Agreement was strongest in the highest tertile of fibrosis burden (mean of differences 16.8% (95% CI = −24.4% to 57.9%, *p* = .433). Fibrosis burden was greater for Atrial‐LGE than Atrial‐EAVM (50.7% ± 10.7% vs. 13.7% ± 7.13%, *p* < .005) for patients in the lowest tertile who were younger, had smaller atria and a greater frequency of paroxysmal atrial fibrillation. Both Atrial EAVM and Atrial LGE were associated with recurrence of arrhythmia following ablation (Atrial‐LGE HR = 1.02 (95% CI = 1.01–1.04), *p* = .047; Atrial‐EAVM HR = 1.02 (95% CI = 1.005–1.03), *p* = .007). A low fibrosis burden (<15%) by Atrial‐EAVM identified patients with very low arrhythmia recurrence. In contrast, a much higher fibrosis burden (>66%) by Atrial‐LGE identified patients failing to respond to ablation.

**Conclusions:**

We demonstrate for the first time that the level of agreement between Atrial‐EAVM and Atrial‐LGE is dependent on the level of atrial cardiomyopathy disease severity. The functional consequences of atrial cardiomyopathy are most evident in patients with the highest anatomical extent of disease.

## Graphical abstract

***Key Question***. Atrail electroanatomic voltage mapping (Atrial‐EAVM) and late gadolinium enhancement imaging (Atrial‐LGE) quantify the functional and anatomic extent of atrial fibrosis, a central component of atrial cardiomyopathy. We aimed to explore the relationships between, and outcomes from, these two contrasting measures.

***Key Finding***. Atrial‐EAVM and Atrial‐LGE correlated with greatest agreement at high‐fibrosis burdens. Atrial‐LGE was higher in patients with low fibrosis burden: these patients were younger and had smaller atria and more paroxysmal atrial fibrillation. Both measures predicted the likelihood of arrhythmia recurrence.

***Take Home Message***. Functional and anatomic measures of atrial fibrosis define the extent of atrial cardiomyopathy. Atrial‐LGE may detect earlier‐stage disease than Atrial‐EAVM, suggesting functional abnormalities only manifest with higher anatomic fibrosis burden. Increasing fibrosis burden determined by either approach predicts arrhythmia recurrence.

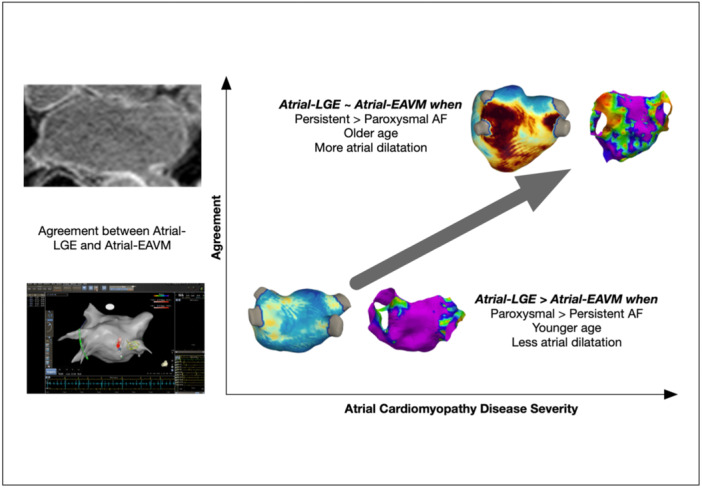

## 1 INTRODUCTION

Atrial cardiomyopathy is defined as the combination of structural, contractile or electrophysiological changes affecting the atria, with the potential for adverse consequences including onset of atrial fibrillation and elevated thromboembolic risk.^1^ Atrial fibrosis is a central component of atrial cardiomyopathy^2^ and is associated with reduced conduction velocity, increased conduction heterogeneity and increased likelihood of unidirectional conduction block.^3^ Fibrosis is therefore a major contributor to atrial fibrillation perpetuation^4^ and treatment failure following ablation.^5^

Two contemporary techniques are available for atrial cardiomyopathy quantification: a functional measure using invasive atrial electroanatomic voltage mapping (Atrial‐EAVM) and an anatomical measure using late gadolinium enhancement (Atrial‐LGE) cardiac magnetic resonance imaging. Atrial‐EAVM identifies atrial regions with recorded voltages below a predefined amplitude, whilst Atrial‐LGE identifies atrial regions with LGE signal intensities above a predefined value.

Quantification of atrial cardiomyopathy disease severity has been applied or is undergoing study, in three main clinical contexts, but with conflicting results: (1) for predicting response to ablation,^5^ (2) for identifying ablation targets,^6^ and (3) for stratification of systemic thromboembolism risk in patients with^7^ and without^8^ atrial fibrillation. However, previous studies have disagreed about the relationship between Atrial‐EAVM and Atrial‐LGE,^9^, ^10^, ^11^, ^12^, ^13^, ^14^ with several studies identifying a positive correlation^10^, ^11^, ^12^ which has not been reproduced in other studies.^9^, ^13^, ^14^

In this context, the clinical role of both techniques is debated. Here, we aimed to compare Atrial‐EAVM and Atrial‐LGE in a large cohort of patients undergoing atrial fibrillation ablation. We sought to explore the relationships between, and outcomes from, these two contrasting measures of atrial cardiomyopathy to understand the factors promoting the conflicting findings of previous studies.

## 2 METHODS

### 2.1 Patient population

Consecutive patients attending for first‐time ablation were screened from 1 January 2016 to 31 December 2018 and included if both preprocedure cardiac magnetic resonance imaging and intra‐procedural electroanatomic voltage mapping were performed. Exclusion criteria were congenital heart disease, previous cardiac surgery, previous left atrial ablation, or inadequate image quality. The study conformed to good clinical practice and was conducted in accordance with the declaration of Helsinki. All data were collected during routine care. The need for written consent was waived following Health Research Authority review (18/HRA/0083).

### 2.2 Atrial cardiac magnetic resonance imaging

Cardiac magnetic resonance imaging was performed on 1.5 T Ingenia (Philips Healthcare, Best) and Aera Magnetom (Siemens, Erlangen) scanners.^15^ A contrast‐enhanced, respiratory navigated (acceptance window ±2.5 mm), ECG trigged magnetic resonance angiogram sequence of the left atrium was acquired 90 s after administration of 0.2 mL/kg Gadovist (Bayer Healthcare Pharmaceuticals) in axial orientation. Imaging was timed to atrial diastole, immediately before mitral valve opening with a duration <100 ms to minimize motion artifact. LGE imaging was performed 20 min after contrast administration using an ECG‐triggered, respiratory navigated inversion recovery spoiled gradient echo sequence in axial orientation (spatial resolution 1.3 × 1.3 × 4 mm^3^ reconstructed to 1.3 × 1.3 × 2 mm^3^, TR 4 ms, TE 2 ms, flip angle 20°, phase encoding direction anterior‐posterior, frequency encoding direction left–right). A preceding single slice multiphase inversion time mapping sequence was performed to determine the correct inversion time to null ventricular myocardium.

### 2.3 Left atrial voltage mapping

All procedures were performed under general anesthesia. A decapolar catheter (St. Jude Medical) was positioned in the coronary sinus. Following trans‐septal puncture, two 8.5 Fr sheaths and a PentaRay multispline mapping catheter (1 mm electrodes, 2‐6‐2 mm spacing) or a Lasso circular mapping catheter (1 mm electrodes, 2‐6‐2 mm spacing) (Biosense Webster, Diamond Bar) were advanced into the left atrium. Patients attending in atrial fibrillation underwent external cardioversion. Voltage maps were created during proximal coronary sinus pacing using Carto3 (Biosense Webster) at a constant cycle length of 500 or 600 ms. Cases with a point density of <1/cm^2^ were excluded.

### 2.4 Data analysis

Categorical assessment of MRI imaging was performed by two reviewers blinded to quantitative analysis findings and according to a standard protocol (see Supplementary Material). Categorical assessment of voltage mapping was performed by dividing patients into quartiles of low‐voltage area.

Quantitative assessment of MRI imaging was performed using CEMRGapp (version 1.0, www.cemrg.com). The inter‐ and intra‐observer reproducibility of this method has previously been shown to be excellent, (ICC 0.88 and ICC 0.94, respectively).^16^ A 3D segmentation of the left atrium, excluding the pulmonary veins, appendage and mitral valve, was generated using the CE‐MRA image and registered to the Atrial LGE image. The left atrial blood pool signal mean and standard deviation were used as reference values to define fibrotic tissue using previously published thresholds: (1) signal intensity greater than 3.3 standard deviations above the blood pool mean^17^; (2) image intensity ratio (IIR) 1.2 x blood pool mean^11^; (3) IIR 1.32 x blood pool mean^11^; and (4) IIR 0.97 x blood pool mean.^18^ The surface area of enhanced tissue was indexed to total left atrial surface area to calculate the fibrosis burden at each threshold. Chamber mean signal intensity was normalized to the blood pool mean as a ratio.

Quantitative analysis of electroanatomic voltage mapping was performed using OpenEP (v1.0.03, https://openep.io).^19^ Each electrogram was reviewed manually and assessed for inclusion based on previously described criteria.^20^ Pulmonary venous and appendage regions were removed using mapping system tools. Any points greater than 7 mm from the atrial surface were removed. Voltage maps were quantified following interpolation in OpenEP using the scattered interpolant method with a fill threshold of 12 mm. Low voltage tissue was defined using three previously published thresholds: <0.5 mV,^21^ <1.17 mV,^22^ and <1.3 mV.^23^ The low voltage area was indexed to the left atrial surface area to calculate the low voltage burden.

For regional analysis, the left atrium was segmented into anterior, roof, posterior, inferior and septal components.^19^ The segmentation method is automatic and objective, based on anatomical landmarks. Code for segmental analysis is available in OpenEP. The pulmonary venous antra were considered as a single entity.

### 2.5 Outcome measures

Clinical follow‐up was performed at 3‐ and 12‐months with 24‐h and 12‐lead ECG recordings or more regularly as required according to clinical status and patient symptoms. For time‐to‐event analysis, the time of atrial fibrillation recurrence was defined as the earliest of the first period of documented atrial fibrillation >30 s or patient‐reported recurrence of typical symptoms, corroborated by subsequent ECG evidence, after a 90‐day blanking period. For the assessment of outcome measures, patients were considered in tertiles of overall fibrosis burden, with the first tertile representing the lowest fibrosis burden and overall fibrosis burden calculated as the mean value of both Atrial‐LGE and Atrial‐EAVM.

### 2.6 Statistical analysis

Normally distributed continuous variables are presented as mean ± standard deviation. Nonnormally distributed or noncontinuous, ordinal data are presented as median (interquartile range). Categorical data are presented as percentages. Relationships between tissue characterization metrics were quantified with Pearson correlation coefficients. Mann Whitney U‐test or Student's *t*‐test were used to compare group medians or means respectively. Two‐sided *p* < .05 was considered statistically significant. Primary analyses for logistic regression were performed with censoring at 1 year or the date of the last contact for patients lost to follow‐up before 1 year. Multivariable analysis was performed using characteristics with *p* < .2 in univariable analysis. Cox proportional hazards was used to assess time‐to‐event analyses and proportionality assumptions met with an assessment of Schoenfeld residuals. Kaplan–Meier curves were compared using log‐rank tests. Statistical analysis was performed using R (version 4.0.2, R Foundation for Statistical Computing, Vienna).

## 3 RESULTS

### 3.1 Patient characteristics

**Figure 1:**
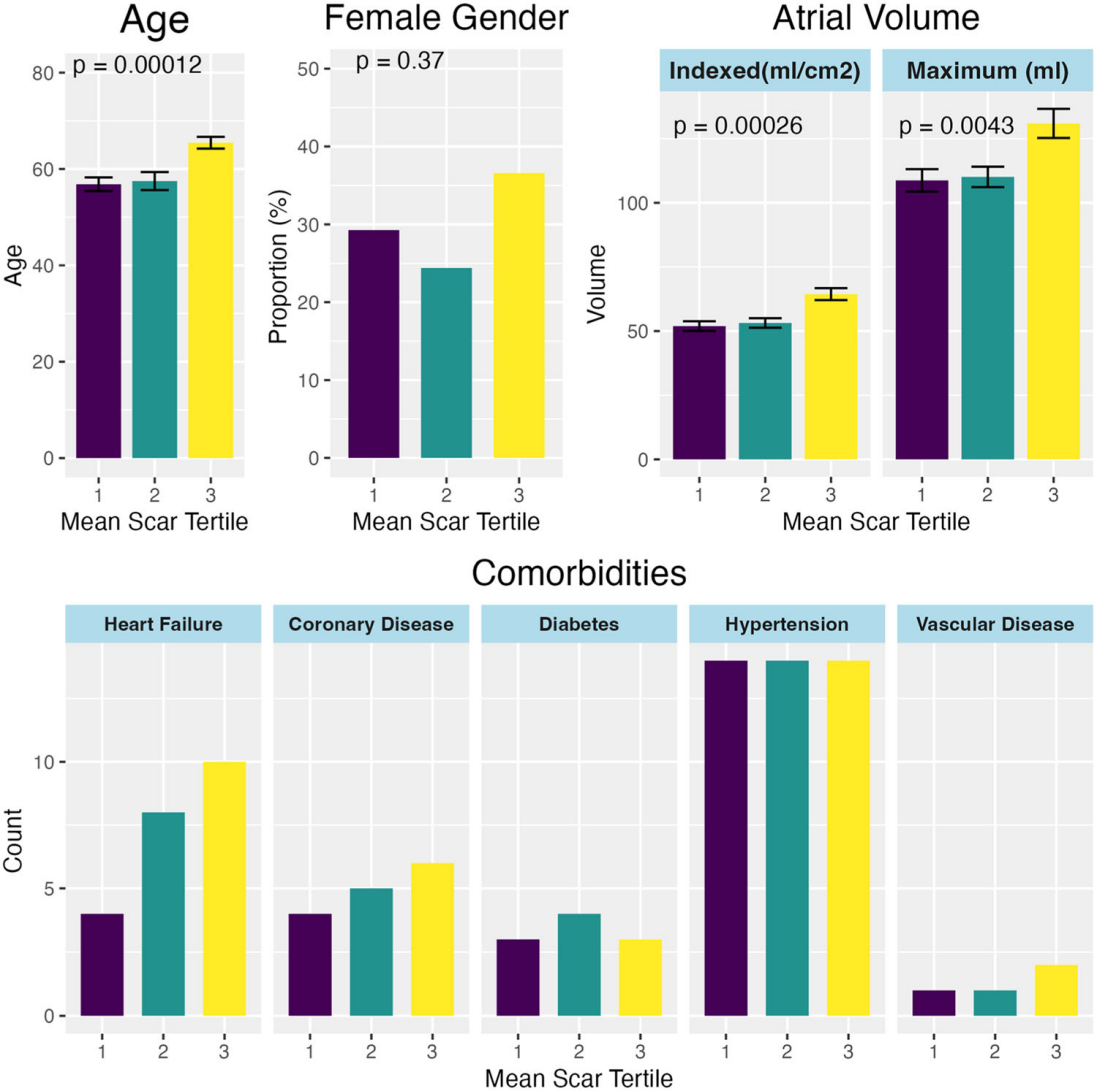
Clinical characteristics of patient sub‐groups defined using tertiles of mean fibrosis burden. CAD, coronary artery disease; DM, diabetes mellitus; HF, heart failure; HTN, hypertension; PVD, peripheral vascular disease.

During the study period, 204 patients met the inclusion/exclusion criteria. In total, 81 patients were excluded from analysis, comprising 33 for inadequate image quality, 32 as voltage mapping was not performed and 16 as voltage mapping was not performed during coronary sinus pacing or did not meet the predefined quality metrics. Atrial‐EAVM and Atrial‐LGE imaging were performed in 123 patients (Table 1). The median±IQR time from imaging to mapping was 72 ± 126 days. All patients underwent pulmonary vein isolation and 53% underwent additional ablation, including mitral isthmus (7%), roof line (2%), cavotricuspid isthmus (16%), fractionated electrogram (2%), or posterior box (49%) ablation. Following electrogram review, the mean number of points remaining was 757 ± 405 per case. The mean point density was 6.6 ± 3.7 points/cm^2^. Patients with the overall lowest fibrosis burden were younger and had lower left atrial volumes but there were no significant differences in the frequency of diabetes mellitus, coronary artery disease, heart failure or hypertension (Figure 1). Patients within the highest tertile of overall fibrosis were more likely to have additional ablation performed (proportion of patients having additional ablation, 26.8% vs. 39.0% vs. 68.3%, *p* < .001 for the first, second, and third tertiles of scar burden respectively).

**Table 1:** Baseline patient characteristics.

	Paroxysmal atrial fibrillation (*n* = 58)	Persistent atrial fibrillation (*n* = 65)	*p*	Total (*n *= 123)
Age (years)	59.4 ± 9.8	60.3 ± 11.1	.530	59.9 ± 10.5
Female gender (*n* [%])	20 (34.5%)	17 (26.2%)	.315	37 (30.1%)
Diabetes (*n* [%])	4 (6.9%)	6 (9.2%)	.636	10 (8.1%)
Coronary disease (*n* [%])	6 (10.3%)	9 (13.8%)	.554	15 (12.2%)
BMI (kg/m^2^)	27.9 ± 4.4	29.8 ± 5.6	.043	28.9 ± 5.2
CHA_2_DS_2_VaSc Median (Q1, Q3)	1.0 (0.25, 2.0)	2.0 (1.0, 2.0)	.440	1.0 (1.0, 2.0)
Hypertension (*n* [%])	17 (29.3%)	25 (38.5%)	.285	42 (34.1%)
Maximum atrial volume (ml)	102.5 ± 25.5	129.2 ± 31.8	< .001	116.6 ± 31.8
Indexed atrial volume (ml)	50.8 ± 11.6	61.5 ± 14.3	< .001	56.5 ± 14.1
LVEF (%)	61.1 ± 7.6	53.8 ± 9.9	< .001	57.3 ± 9.6
AAD before ablation (*n* [%])	28 (48.3%)	23 (35.4%)	.147	51 (41.5%)

Abbreviations: AAD, antiarrhythmic drugs; BMI, body mass index; CHA_2_DS_2_VaSc, congestive heart failure, hypertension, age, diabetes mellitus, stroke, vascular disease and sex category score; LVEF, left ventricular ejection fraction.

### 3.2 Categorical assessment of fibrosis

**Figure 2:**
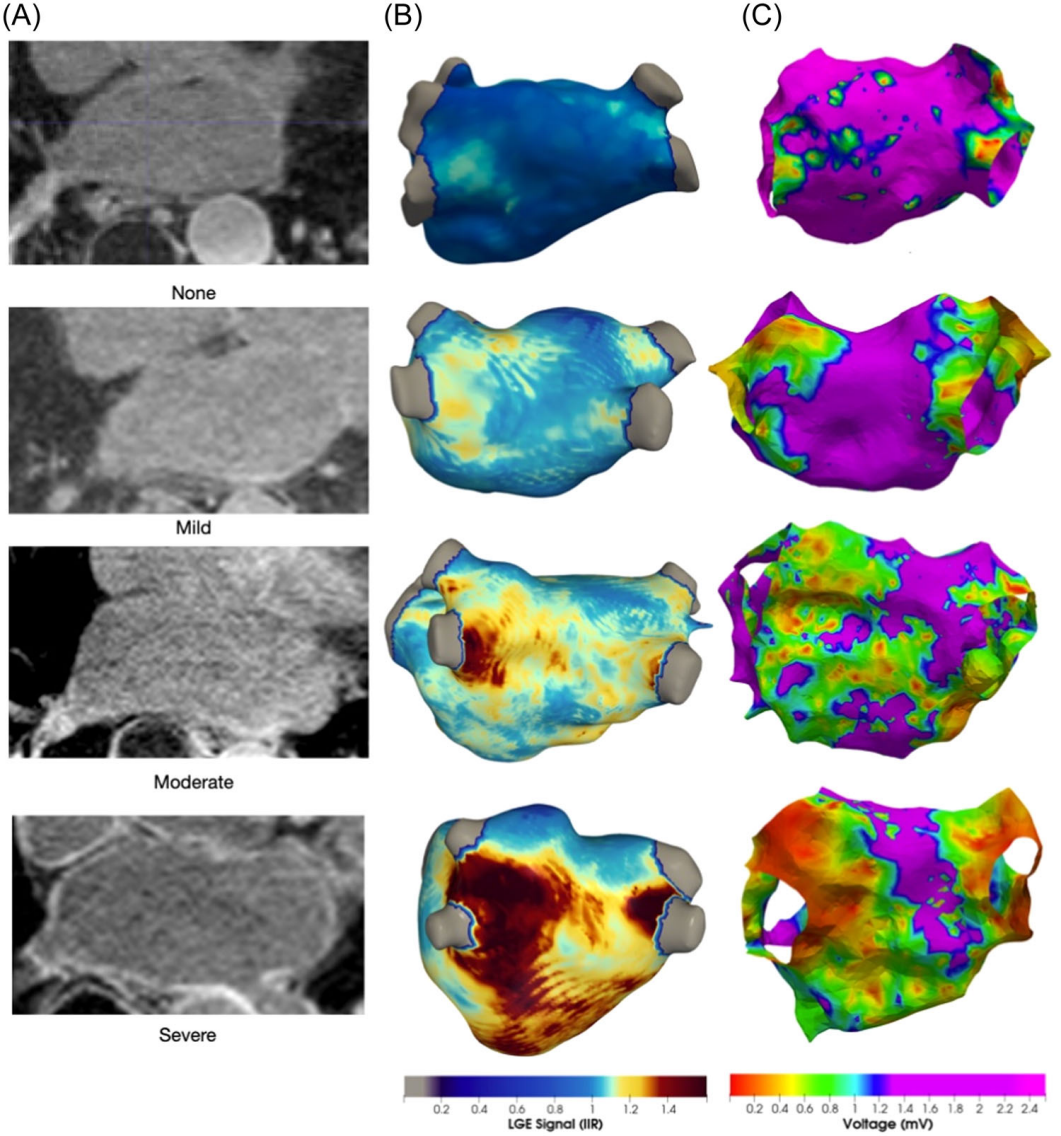
Example Atrial LGE images and electroanatomic voltage maps. (A) Atrial LGE appearances for each category of qualitative assessment (B) Atrial LGE shells reconstructed from Atrial LGE imaging and visualized using CEMRGapp. (C) Electroanatomic voltage shells reconstructed and visualized using OpenEP. LGE, late gadolinium enhancement.

**Figure 3:**
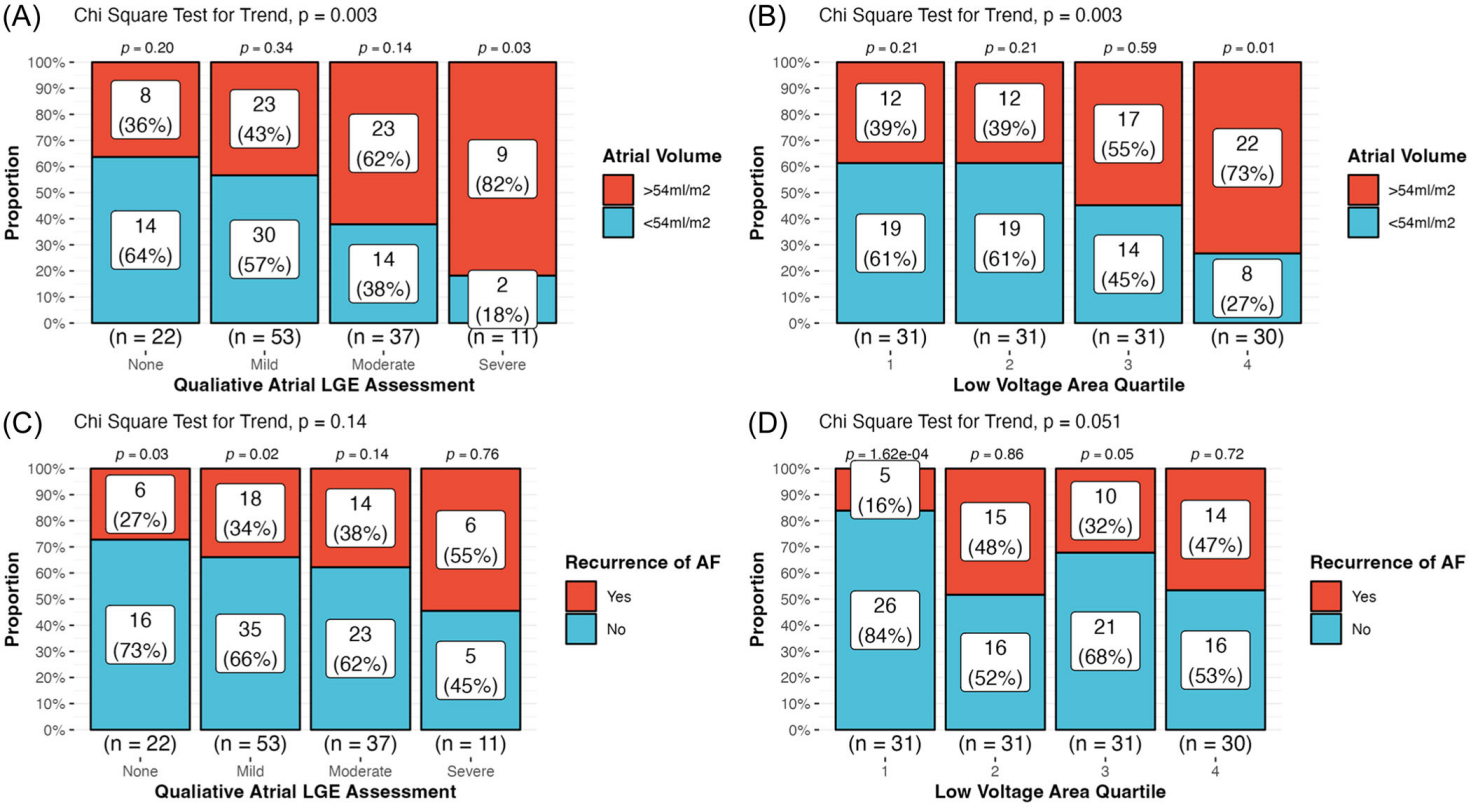
Qualitative analysis of Atrial‐LGE and Atrial‐EAVM. (A) Prevalence of atrial dilation increases as visual severity category of Atrial LGE increases. (B) Prevalence of atrial dilatation increases with increasing quartiles of low voltage (C) AF recurrence increases with increasing visual Atrial‐LGE category. (D) AF recurrence is low in the absence of significant low voltage but increases significantly in the presence of any low voltage area (quartile 2). EAVM, electroanatomic voltage mapping; LGE, late gadolinium enhancement.

Categorical analysis of Atrial‐LGE identified 22 patients with no fibrosis, 53 patients with mild fibrosis, 37 patients with moderate fibrosis, and 11 patients with severe fibrosis (Figure 2). The prevalence of atrial dilatation increased significantly with increasing severity category of Atrial‐LGE (*p *= .0034) (Figure 3A). A similar relationship was also seen between atrial low voltage category and atrial dilatation (*p *= .0031) (Figure 3B).

The prevalence of AF recurrence increased gradually across the Atrial‐LGE severity categories: in the lowest severity categories (i.e., “none” or “mild” fibrosis) significantly more patients had no recurrence than recurrence (*p *= .03 and *p*= .02) whilst in the highest severity categories there was a more even split between patients with and without recurrence (*p *= .14 and *p*= .76). In contrast, for Atrial‐EAVM, there was an abrupt increase in the proportion of patients with recurrence between the first two categories of Atrial‐EAVM (5/26 patients vs. 15/16 patients, *p*= .0066), indicating that the presence of only a low burden of atrial low voltage was significantly associated with arrhythmia recurrence (Figure 3C,D).

### 3.3 Quantitative assessment of fibrosis

There were positive, statistically significant, correlations between fibrosis burden measured using Atrial‐EAVM and Atrial‐LGE for all combinations of established thresholds (Figure S1). The strongest relationships were seen when Atrial‐LGE was quantified using the >3.3 SD and IIR > 0.97 thresholds. However, visual analysis of scatter and Bland–Altman plots showed significant outlying data contributing to this relationship for correlations involving Atrial‐LGE 3.3 SD (Figure S2). Regression analysis including Q–Q plots and residual versus leverage plots, confirmed a series of outliers contributing to the relationship between Atrial‐EAVM (all thresholds) and Atrial‐LGE 3.3 SD (Figure S3).

Quantitative analysis was therefore performed using Atrial‐LGE IIR > 0.97 and Atrial‐EAVM LVA < 1.17 mV as the thresholds yielding the strongest robust relationship between Atrial‐LGE and Atrial‐EAVM. Using these thresholds, quantitative analysis demonstrated a mean fibrosis burden of 32.4% ± 21.8% for Atrial‐EAVM and 62.0% ± 14.3% for Atrial‐LGE, with an overall average fibrosis burden 46.9% ± 15.3%.

**Figure 4:**
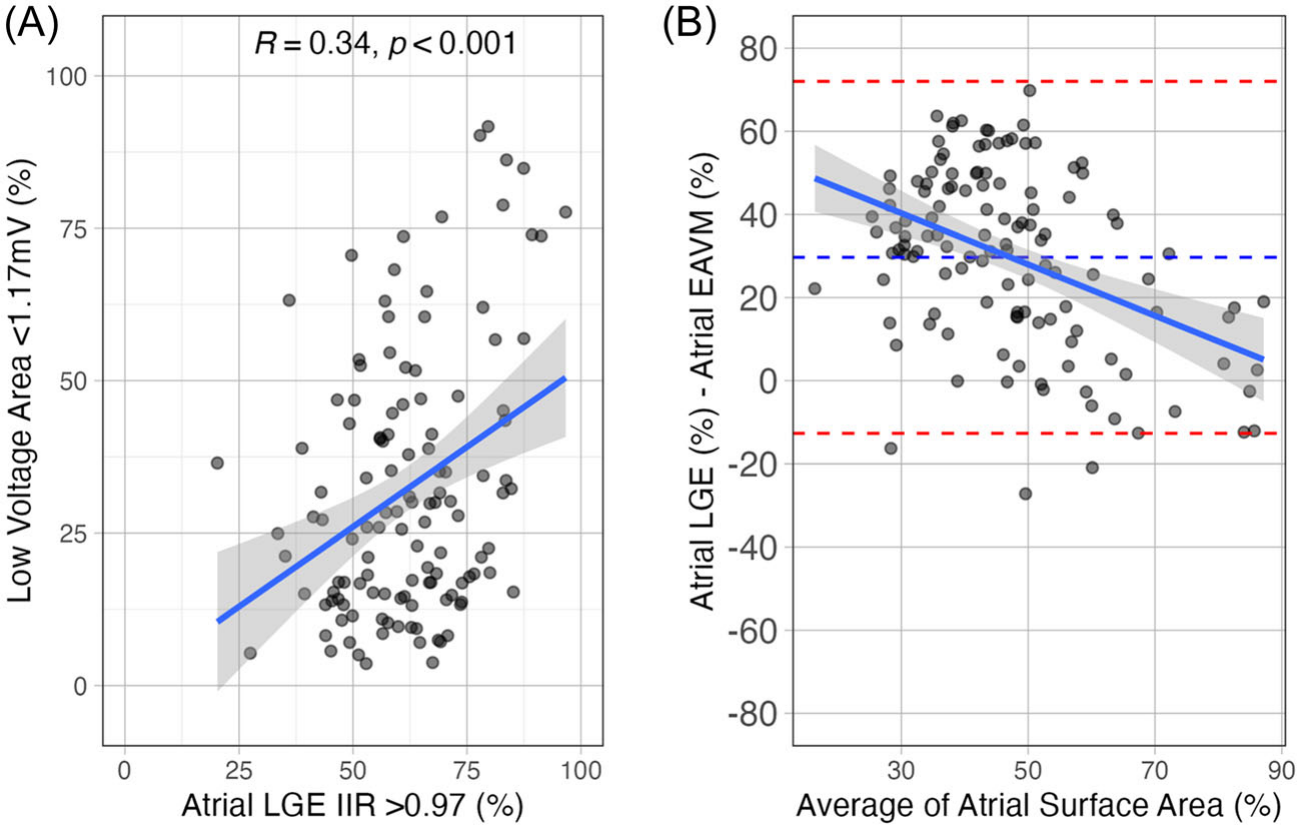
Comparison between Atrial‐EAVM and Atrial‐LGE. (A) Linear regression with 95% confidence intervals. R values are Pearson correlation coefficients. (B) Bland–Altman plot showing agreement between the atrial area identified as abnormal by cardiac magnetic resonance imaging (image intensity ratio [IIR] > 0.97 threshold) and the atrial area identified as abnormal by electroanatomic voltage mapping (low voltage area <1.17 mV threshold). EAVM, electroanatomic voltage mapping; LGE, late gadolinium enhancement.

**Figure 5:**
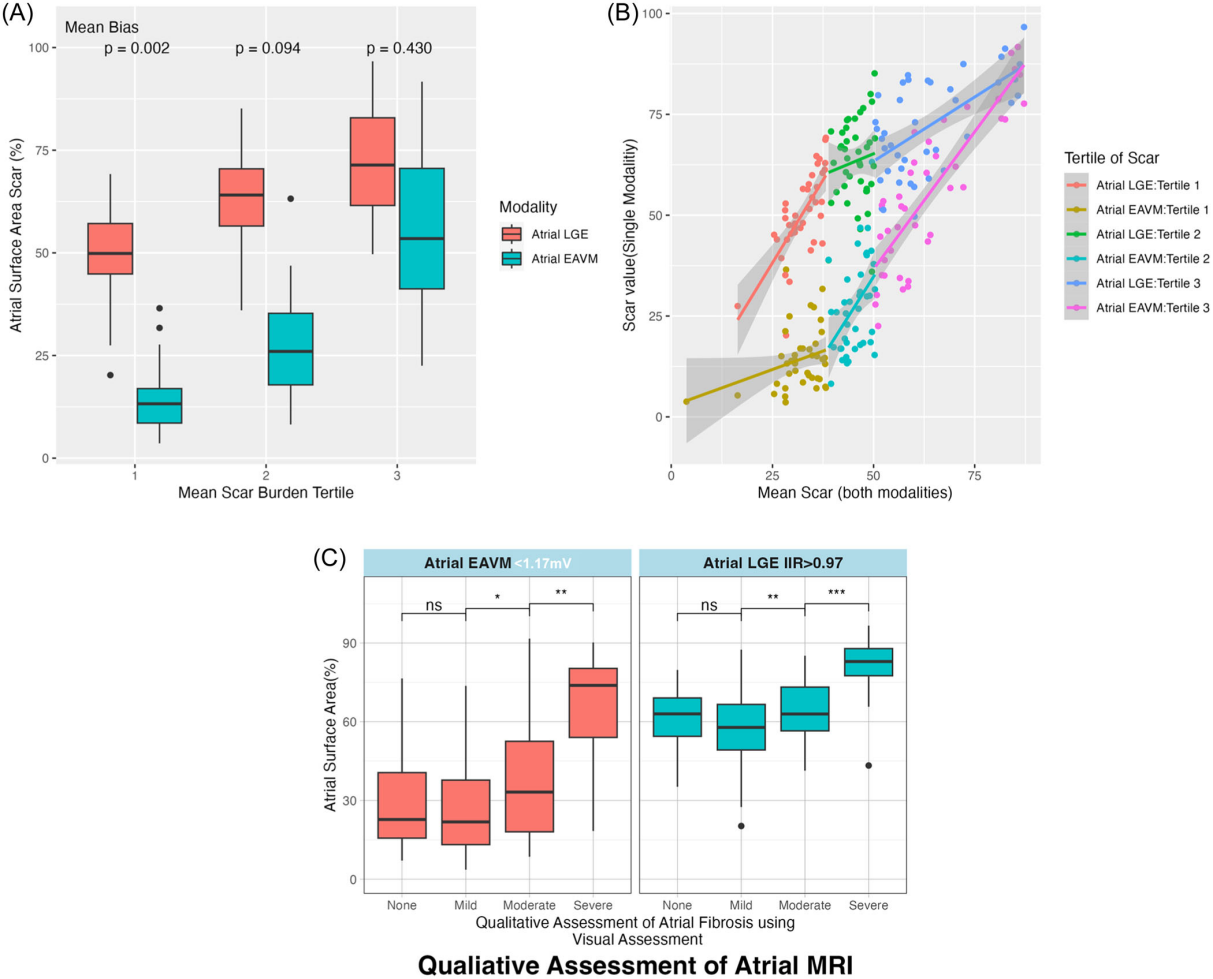
(A) Median atrial surface area identified as fibrotic by each modality, showing reducing absolute differences between the modalities with increasing overall fibrosis burden. *p* values represent tests of significance for between‐modality differences in disease severity quantification at each tertile of overall fibrosis burden. (B) Decreasing differences between modalities is shown for each tertile of mean scar (both modalities), with convergence of regression lines at the highest fibrosis burden. (C) Qualitative assessment of Atrial‐LGE and the relationship to quantitative fibrosis area, showing that for both Atrial‐LGE and Atrial‐EAVM, a higher visual grade of MRI LGE is associated with increasing fibrosis burden. EAVM, electroanatomic voltage mapping; LGE, late gadolinium enhancement.

Bland Altman assessment of bias showed that compared with Atrial‐EAVM, atrial fibrosis burden quantified by Atrial‐LGE was 29.4% higher with 95% limits of agreement of 33.2%–25.6% (Figure 4). The mean difference in fibrosis burden decreased with increasing mean fibrosis burden (R^2^ = 0.18, *p*< .005). Disease severity quantified by Atrial‐LGE was therefore greater than that quantified by Atrial‐EAVM for patients in the lowest tertiles of overall fibrosis burden, but this difference between modalities decreased with increasing disease severity for patients in the middle and highest tertiles of overall fibrosis burden (Table 2, Figure 5A,B). There were no significant differences in Atrial‐LGE between patients scanned in AF or sinus rhythm in any of the tertiles of fibrosis burden (*p* = .056, *p* = .78, and *p* = .24).

**Table 2:** Fibrosis burden tertiles.

Tertile	Overall fibrosis burden	Mean difference	95% Limits of agreement	*p* value (Welch's *t*‐test)
1	0%–38.6%	35.8%	4.6% to 67.0%	.002
2	38.8%–50.3%	35.8%	−6.13% to 77.8%	.094
3	50.4%–87.1%	16.8%	−24.4% to 57.9%	.433

*Note*: Mean differences and 95% limits of agreement for patient subgroups, defined using tertiles of mean fibrosis burden. *p* values represent tests of significance for between‐modality differences in disease severity quantification at each tertile of overall fibrosis burden. Significant bias exists for the lower two tertiles but it non significant for the highest tertile of patients.

Disease severity quantified by both Atrial‐LGE and Atrial‐EAVM increased significantly across visual categories of MRI assessment: patients assessed as visually severe fibrosis on MRI had both greater quantified LGE and greater quantified low voltage area than patients in lower categories of visual fibrosis assessment (Figure 5C).

**Figure 6:**
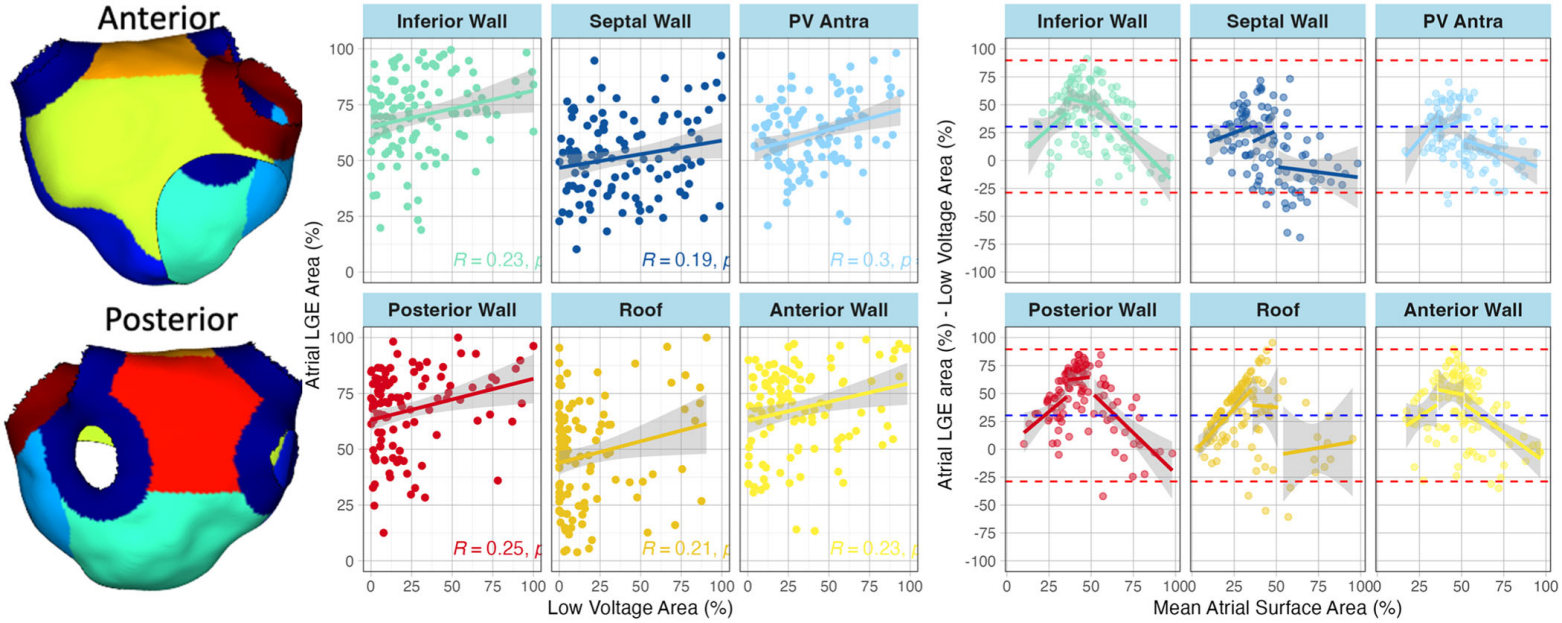
The relationship between Atrial‐LGE and Atrial‐EAVM for each segment of the atria. The relationship is modest or weak for all segments. The strongest is within the PV antra, the weakest within the septum. Within the roof and posterior wall there appears to be significantly more Atrial‐LGE fibrosis than Atrial‐EAVM fibrosis with a large number of zero values using voltage mapping. EAVM, electroanatomic voltage mapping; LGE, late gadolinium enhancement.

Regional correlations between Atrial‐LGE and Atrial‐EAVM were modest. The strongest correlation between modalities was found in the PV antra, whereas the lowest correlation existed in the septum. (Figure 6). Assessment of bias within each segment showed similar findings to the overall atria, that is, reduced bias in the highest tertile of overall fibrosis burden.

### 3.4 AF classification

Atrial fibrosis burden measured using Atrial‐EAVM was greater in patients with persistent atrial fibrillation (Table 3). Consistent with this, mean left atrial voltage was lower in patients with persistent atrial fibrillation (1.73 ± 0.63 vs. 2.08 ± 0.57 mV, *p* < .001). In contrast, neither fibrosis burden measured using Atrial‐LGE or mean LGE signal intensity showed significant differences between patients classified with paroxysmal or persistent atrial fibrillation.

**Table 3:** Atrial tissue characterization, atrial fibrillation type, and recurrence.

	Atrial fibrillation type			Arrhythmia recurrence		
	Paroxysmal atrial fibrillation (*n *= 58)	Persistent atrial fibrillation (*n *= 65)	*p*	No (*n *= 79)	Yes (*n *= 44)	*p*
Fibrosis burden measured by Atrial‐EAVM (%)	26.3 ± 18.0	37.8 ± 23.6	.005*	28.5 ± 20.2	39.3 ± 23.1	.004*
Mean left atrial voltage (mV)	2.08 ± 0.57	1.73 ± 0.63	.001*	2.02 ± 0.64	1.67 ± 0.55	.006*
Fibrosis burden measured by Atrial‐LGE (%)	60.478 ± 13.8	63.377 ± 14.6	.264	60.169 ± 13.8	65.308 ± 14.6	.074
Mean signal intensity (IIR)	1.004 mV ± 0.06	1.012 mV ± 0.07	.530	1.003 (0.064)	1.018 (0.071)	.196

*Note*: Comparison between electroanatomic mapping and CMR tissue characterization metrics for each of the clinical outcomes. Fibrosis burden quantified using Atrial‐EAVM at 1.17 mV threshold. Fibrosis burden quantified using Atrial‐LGE at IIR 0.97 threshold.

Abbreviations: EAVM, electroanatomic voltage mapping; LGE, late gadolinium enhancement.

### 3.5 Recurrence of arrhythmia

The mean follow‐up time was 626 ± 323 days. Over this follow‐up period, atrial fibrillation recurred in 44 (36%) patients: 16 participants with paroxysmal and 27 with persistent atrial fibrillation. In patients with arrhythmia recurrence in the first year of follow up, atrial fibrosis burden measured using Atrial‐EAVM was greater (39.32 ± 23.10 vs. 28.48% ± 20.20%, *p*= .004) and mean left atrial voltage was lower (1.67 ± 0.55 vs. 2.02 ± 0.64 mV, *p*= .006). Atrial fibrosis burden measured using Atrial‐LGE was higher for patients with arrhythmia recurrence (65.3 ± 14.5 vs. 60.2% ± 13.8%, *p*= .074) but this difference did not reach statistical significance (Table 3).

In univariable Cox proportional hazards analyses, over the first year of follow‐up, there were significant associations between recurrence of atrial fibrillation for both increased Atrial LGE (hazard ratio [HR] 1.02 [95% CI = 1.01–1.04]), *p* = .047), and increased Atrial EAVM (HR 1.02 [95% CI0 = 1.005–1.03]), *p* = .007). In multivariable analysis, (adjusting for variables with *p* < .2 in univariable analysis, indexed left atrial volume only) Atrial‐LGE (HR = 1.02, confidence interval 0.99–1.05, *p* = .051) and Atrial‐EAVM (HR 1.03, confidence interval 1.00–1.03, *p* = .06) yielded similar hazard ratios for risk of arrhythmia recurrence (Table 4). The inclusion of further ablation lesions beyond pulmonary vein isolation in the model neither changed the relationship between Atrial‐LGE/Atrial‐EAVM and arrhythmia recurrence nor was not associated with arrhythmia recurrence itself (HR = 1.4, Confidence Interval 0.78–2.2, *p* = .24).

**Table 4:** Univariable analysis for prediction of atrial fibrillation recurrence following ablation.

	No recurrence of arrhythmia (*n* = 79)	Recurrence of arrhythmia (*n* = 44)	Hazard ratio (95% confidence interval)	*p*
Age (years)	59.2 ± 10.2	61.2 ± 10.8	1.02 (0.98–1)	.33
Female gender (*n* [%])	24 (64.9)	13 (35.1)	1.14 (0.59–2.2)	.70
Persistent atrial fibrillation (*n* [%])	38 (58.5)	27 (41.5)	1.45 (0.79–2.7)	.22
Indexed left atrial volume (ml)	**54.5** ± **13.7**	**60.1** ± **14.3**	**56.5** ± **14.1**	.03
LVEF (%)	57.7 ± 9.5	56.4 ± 9.8	0.989 (0.96–1)	.48
Diabetes (*n* [%])	8 (80.0)	2 (20.0)	0.448 (0.11–1.9)	.28
BMI (kg/m^2^)	29.2 ± 5.2	28.4 ± 5.0	0.963 (0.91–1)	.22
Hypertension (*n* [%])	24 (57.1)	18 (42.9)	1.33 (0.73–2.4)	.36
Fibrosis burden measured by Atrial‐EAVM (%)	**28.5** ± **20.2**	**39.3** ± **23.1**	**1.02 (1–1)**	.01
Fibrosis burden measured by Atrial‐LGE (%)	**60.2** ± **13.8**	**65.3** ± **14.6**	**1.02 (1–1)**	.04

*Note*: Hazard ratios, using acox proportional hazards model are shown for a single unit increase in each parameter. Fibrosis burden quantified using Atrial‐EAVM at 1.17 mV threshold. Fibrosis burden quantified using Atrial‐LGE at IIR 0.97 threshold. Statistically significant *p* values shown in bold.

Abbreviations: BMI; body mass index; EAVM; electroanatomic voltage mapping; LGE; late gadolinium enhancement; LVEF; left ventricular ejection fraction.

**Figure 7:**
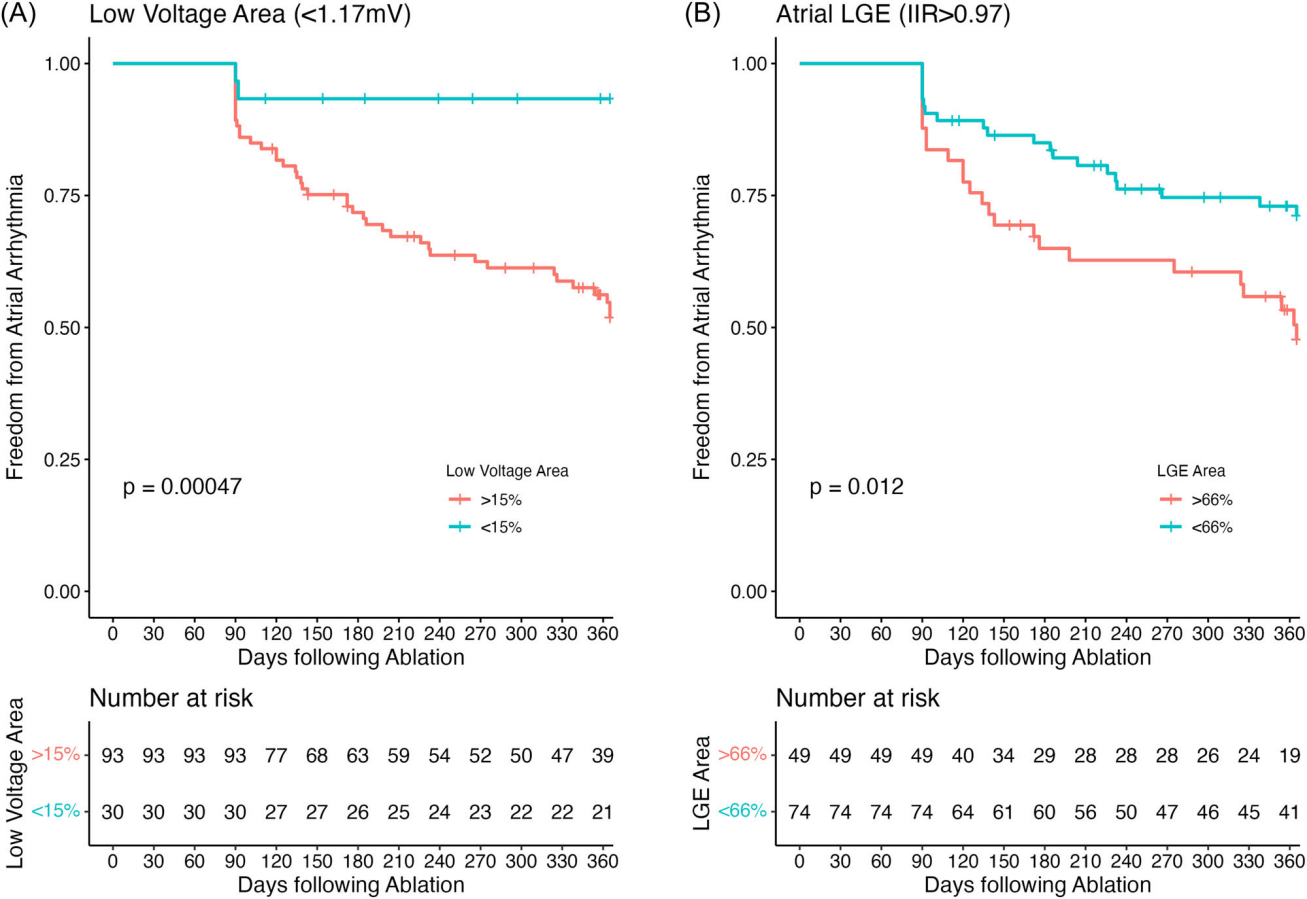
Time to atrial fibrillation recurrence. (A) Time to recurrence categorizing patients using Atrial‐LGE. (B) Time to recurrence categorizing patients using Atrial‐EAVM. *p* values from log‐rank tests. Optimal split thresholds are shown for each modality. EAVM, electroanatomic voltage mapping; LGE, late gadolinium enhancement.

Kaplan–Meier analysis revealed the probability of arrhythmia recurrence was significantly greater in patients with a high fibrosis burden for both Atrial LGE (*p* = .012) and Atrial EAVM (*p* < .001). A low fibrosis burden (<15%) quantified by Atrial EAVM identified a population of patients with very low arrhythmia recurrence during follow‐up. In contrast, a much higher fibrosis burden (>66%) quantified by Atrial LGE identified a population of patients failing to respond to ablation, with a similar 1‐year recurrence rate as those patients with only a low burden of atrial low voltage (freedom from arrhythmia, Atrial‐EAVM fibrosis >15% 39/93 vs. Atrial‐LGE fibrosis >66% 19/49, chis square *p* = .84) (Figure 7).

## 4 DISCUSSION

In the context of conflicting prior studies, we sought to explore and understand the relationship between Atrial‐LGE (an anatomical measure of atrial fibrosis) and Atrial‐EAVM (a functional measure of the consequences of atrial fibrosis). We demonstrate for the first time that the level of agreement between Atrial‐LGE and Atrial‐EAVM is dependent on the level of atrial cardiomyopathy disease severity. We show that Atrial‐LGE identifies greater fibrosis in younger patients with smaller atrial and paroxysmal atrial fibrillation, which may represent an earlier stage of atrial cardiomyopathy. Finally, we show that disease severity quantified by both Atrial‐LGE and Atrial‐EAVM is associated with arrhythmia recurrence following catheter ablation of atrial fibrillation, but that modality specific thresholds are required to interpret each modality. This is the largest study to date demonstrating the interrelationship between functional and anatomical measures of atrial fibrosis. The results help to explain the conflicting findings of prior studies examining atrial cardiomyopathy.

### 4.1 Relationship between Atrial‐LGE and Atrial‐EAVM

Previous studies have sought to validate Atrial‐LGE against Atrial‐EAVM^12^ or to identify optimal thresholds for defining abnormal tissue using Atrial‐LGE, using Atrial‐EAVM as the reference standard.^18^, ^24^ Conflicting results have led to criticism of Atrial‐LGE as an assessment technique for atrial cardiomyopathy.^25^ However, cardiac magnetic resonance imaging and electroanatomic mapping are contrasting techniques, with one providing an anatomical assessment of fibrosis and the other providing a representation of the functional consequences of multiple disease processes present in patients with atrial fibrillation.

Correspondingly, we found that the overall correlation between Atrial‐LGE and Atrial‐EAVM was moderate. The strength of the correlation was dependent on the thresholds chosen to define abnormal atrial tissue. The thresholds yielding the most robust correlation were IIR > 0.97 (for Atrial‐LGE) and voltage <1.17 mV (for Atrial‐EAVM). We further undertook qualitative assessment of Atrial‐LGE demonstrating that fibrosis burden quantified by both Atrial‐LGE and Atrial‐EAVM increased with increasing qualitative severity of Atrial‐LGE, blinded to the voltage mapping findings. To our knowledge, this observation is new and supports the assertion that both Atrial‐LGE and Atrial‐EAVM are in part dependent upon related components of atrial cardiomyopathy, that is, atrial fibrosis.

### 4.2 Determinants of Atrial‐LGE and Atrial‐EAVM

We report for the first time that Atrial‐LGE appears to identify atrial cardiomyopathy at an earlier stage in the disease process than Atrial‐EAVM. This conclusion is supported by three observations. First, at low‐fibrosis burdens, atrial cardiomyopathy disease severity quantified by Atrial‐EAVM was underestimated in comparison to Atrial‐LGE whereas there was no systematic bias between modalities at high‐fibrosis burdens. This relationship appears to be true regardless of which segment of the atrial wall is studied. Second, the patient characteristics in the low fibrosis burden tertile were consistent with earlier disease: these patients were younger, with smaller atria and a higher likelihood of paroxysmal atrial fibrillation compared to patients in higher tertiles. Finally, patterns of recurrence of arrhythmia were consistent with Atrial‐LGE identifying earlier disease: the presence of even a low burden of fibrosis, defined by Atrial‐EAVM, was associated with a significantly increased risk of arrhythmia recurrence, whilst a much higher burden of fibrosis defined by Atrial‐LGE was required to indicate increased arrhythmia risk. This latter observation is supported by both quantitative and qualitative assessment.

There are two potential explanations for our observations. First, voltage mapping may be insensitive to early fibrosis. Multiple factors influence recorded contact atrial electrogram voltages,^26^ including wall thickness,^27^ peri‐atrial fat,^28^ autonomic influence^29^ and electrical remodeling during rapid tissue activation.^30^ The relative magnitude of the effect of these factors on voltage is unknown. Alternatively, LGE may overestimate atrial cardiomyopathy in the early stage. Whilst we demonstrate here that this early identification of fibrosis is associated with arrhythmia recurrence, further work is needed to determine whether therapeutic interventions can alter the disease process at this early stage.

Alternatively, the pattern of fibrosis may have differential effects on Atrial‐LGE and Atrial‐EAVM. Fibrosis has been described as perimysial (resulting in separation between myocyte bundles) or endomysial (resulting in separation of myocytes within bundles).^31^ Whilst Atrial‐LGE may detect both types of fibrosis, only endomysial fibrosis has been shown to result in increased complexity of fibrillatory conduction.^32^ Conduction patterns during regular rhythms, such as during paced voltage mapping, may also be differentially influenced by the pattern of fibrosis, although this hypothesis has yet to be tested. The data presented here would be consistent with the occurrence of perimysial followed by endomysial fibrosis (reflected in Atrial‐LGE changes preceding Atrial‐EAVM changes). In a recent study, Maesen et al investigated the effects of total fibrosis (which may be detected by Atrial‐LGE) compared to endomysial fibrosis (which may influence Atrial‐EAVM) on fibrillatory conduction patterns. They reported that endomysial fibrosis, rather than total fibrosis, was the main determinant of conduction disturbances during atrial fibrillation in patients with paroxysmal or longstanding persistent atrial fibrillation.^32^

Finally, we demonstrate that areas of the atria had significantly higher Atrial‐LGE than Atrial‐EAVM in certain segments of the atria. For example, the atrial roof showed significant higher Atrial‐LGE areas with no Atrial‐EAVM fibrosis present. This may be related to the difficulties in assessing areas of the atria wall which sit within, rather than across, nonisotropic imaging slices. However, the imaging sequence has been previously validated and the relatively larger slice thickness aids in maintaining adequate signal to noise ratio within a clinically appropriate imaging duration.^33^ Despite this, even within this segment, the degree of agreement between the two modalities increases with overall higher fibrosis burdens, consistent with the overall study findings.

### 4.3 Comparison to previous studies

Seventeen prior studies have examined the relationship between Atrial‐EAVM and Atrial‐LGE.^34^ However, most studies included patients with postablation atrial scar. Only six studies have presented data on native atrial fibrosis, providing data on a total of 240 patients. In comparison, we now present data on an additional 123 patients which is also more than double the size of any single prior study. Three of the six relevant prior studies reported a positive correlation between Atrial‐EAVM and Atrial‐LGE,^10^, ^11^, ^12^ while the other three did not.^9^, ^13^, ^14^ Drawing direct comparisons between studies is challenging owing to different methodologies. However, it is notable that all three studies reporting a correlation between Atrial‐EAVM and Atrial‐LGE appear to have studied patients with a high degree of fibrosis. For example, Oakes et al. studied 54 patients, approximately one‐half of whom had moderate or extensive atrial enhancement.^10^ Of the patients with mild enhancement, half had >50% enhancement of the posterior wall and one‐third had >50% enhancement of the septum. Benito et al. studied 15 patients with mean IIR 0.98 ± 0.2 in paroxysmal, and 0.97 ± 0.2 in persistent, atrial fibrillation patients.^11^ Based on our methodology, most patients in these studies would be characterized as moderate or severe atrial fibrosis. Caixal et al. reported lower voltages in regions of atrial LGE with IIR > 1.2. This threshold is higher than that used here and therefore likely to identify regions of denser fibrosis.^12^ Conversely, Eichenlaub et al. studied 37 patients but did not find a correlation between Atrial‐EAVM and Atrial‐LGE. However, the median left atrial surface area with low voltage was 12.9% (for a 1.0 mV cut‐off) and 2.7% (for a 0.5 mV cut‐off), indicating that the patients studied in this cohort had early stage atrial cardiomyopathy.^14^ The final two studies which did not show a correlation between Atrial‐EAVM and Atrial‐LGE performed voltage mapping during atrial fibrillation, with chaotic electrical activity likely to lead to reduced voltage values.^9^, ^13^

We can now attempt to explain prior discrepancies in part based on the different degrees of fibrosis amongst the patients included in previous studies: studies showing a correlation between Atrial‐EAVM and Atrial‐LGE included patients with more extensive atrial cardiomyopathy than studies not showing a correlation between Atrial‐EAVM and Atrial‐LGE. Our data indicates that this result is expected, since at low levels of atrial cardiomyopathy Atrial‐LGE identifies more extensive disease than Atrial‐EAVM.

### 4.4 Atrial fibrosis and recurrence after catheter ablation

Atrial‐EAVM and Atrial‐LGE were both associated with recurrence of atrial fibrillation following catheter ablation. Using the Atrial‐LGE threshold of IIR > 0.97, a surface area >66% of the atrium was most effective in differentiating between those with and without recurrence. Using this relatively low threshold for the identification of atrial fibrosis (which identifies a greater area of tissue as abnormal), the surface area best dichotomizing patients into those with or without arrhythmia recurrence is higher than that reported by prior studies.^35^ This lower threshold may detect patchy fibrosis and widespread atrial tissue changes, in addition to areas of contiguous dense scar. Such areas of patchy fibrosis may be critical for arrhythmia maintenance,^36^ given the observation that areas of dense fibrosis are less likely to harbor regions of continuous electrical activity during atrial fibrillation.^37^

A bipolar voltage threshold of <0.5 mV is conventionally used for the characterization of abnormal left atrial tissue during ablation procedures,^27^ with ablation of low voltage regions <0.5 mV recently shown to improve arrhythmia recurrence following catheter ablation.^38^ This threshold was not associated with arrhythmia recurrence in the patients studied here, potentially due to the high proportion of patients without low voltage areas <0.5 mV. In contrast, a higher voltage threshold of <1.17 mV was associated with arrhythmia recurrence, provided the most robust correlation with Atrial‐LGE and revealed the full spectrum of low‐voltage burden present amongst the patients studied (Figure S1). This observation is supported by Yang et al. who described a voltage threshold of 1.3 mV to improve identification of abnormal atrial tissue when using smaller, more closely spaced electrodes, as compared to the lower threshold of 0.5 mV.^39^ The present study therefore provides the important finding that a higher voltage threshold, which is more than double the conventional threshold, is more sensitive for the identification of atrial cardiomyopathy.

## 5 LIMITATIONS

During ablation procedures, all patients underwent pulmonary vein isolation whilst 53% patients underwent additional ablation. This data must therefore be interpreted in relation to procedures involving pulmonary vein isolation alone. Nevertheless, additional ablation to pulmonary vein isolation is frequently used during atrial fibrillation ablation and this work is therefore representative of real‐world practice.

The analysis thresholds for quantifying Atrial‐LGE and Atrial‐EAVM were based on the correlation between modalities. Whilst this approach does not necessarily translate to the optimal sensitivity/specificity for detecting fibrosis, in the absence of a gold‐standard technique to measure fibrosis it provides an unbiased method to investigate the relationships between modalities. It is possible that at higher levels of disease severity, both techniques experience increased signal to noise ratio, however the relationship between the modalities exists regardless of the threshold chosen. A comparison of both techniques at the full range of published thresholds is given in the supplementary material.

Finally, atrial LGE may be limited in its detection of atrial fibrosis by spatial resolution. It is possible that relevant atrial fibrosis exists smaller resolution than is detectable by this sequence. However, voltage mapping is similarly limited by spatial resolution and the ability of small areas of fibrosis to alter atrial electrophysiology is unknown. Higher resolution voltage mapping, enabled through the future availability of multipolar microelectrode catheters may improve the ability of voltage mapping to detect early fibrosis.

Voltage mapping may be performed during pacing (as done here), sinus rhythm and during AF. Whether the observations made here extend to maps created during sinus rhythm, or indeed with alternative catheter/electrode configurations is uncertain.

## 6 CONCLUSIONS

Atrial‐EAVM and Atrial‐LGE are complementary rather than equivalent techniques for the assessment of atrial cardiomyopathy disease severity. We present the largest study to date of patients undergoing assessment with both techniques, demonstrating for the first time that the level of agreement between Atrial‐LGE and Atrial‐EAVM is dependent on the level of atrial cardiomyopathy disease severity. Whilst Atrial‐LGE can identify earlier stage atrial cardiomyopathy, both Atrial‐LGE and Atrial‐EAVM are associated with arrhythmia recurrence following catheter ablation of atrial fibrillation. These results help to explain the conflicting findings of prior studies and will permit the design of future studies using atrial cardiomyopathy assessment to evaluate therapies and predict treatment outcomes.

## Supporting information

Supporting information.

Supporting information.

## Data Availability

The data that support the findings of this study are available on request from the corresponding author. The data are not publicly available due to privacy or ethical restrictions.
