# Supplementary material for: Quantification of atrial cardiomyopathy disease severity by electroanatomic voltage mapping and cardiac magnetic resonance imaging
Source: J Cardiovasc Electrophysiol. 2024 Dec 30;36(2):467–79. doi: 10.1111/jce.16462 (PMC11837893; doi:10.1111/jce.16462)
Supplement: Supplementary file 1 — Supporting information. [file JCE-36-467-s002.docx]

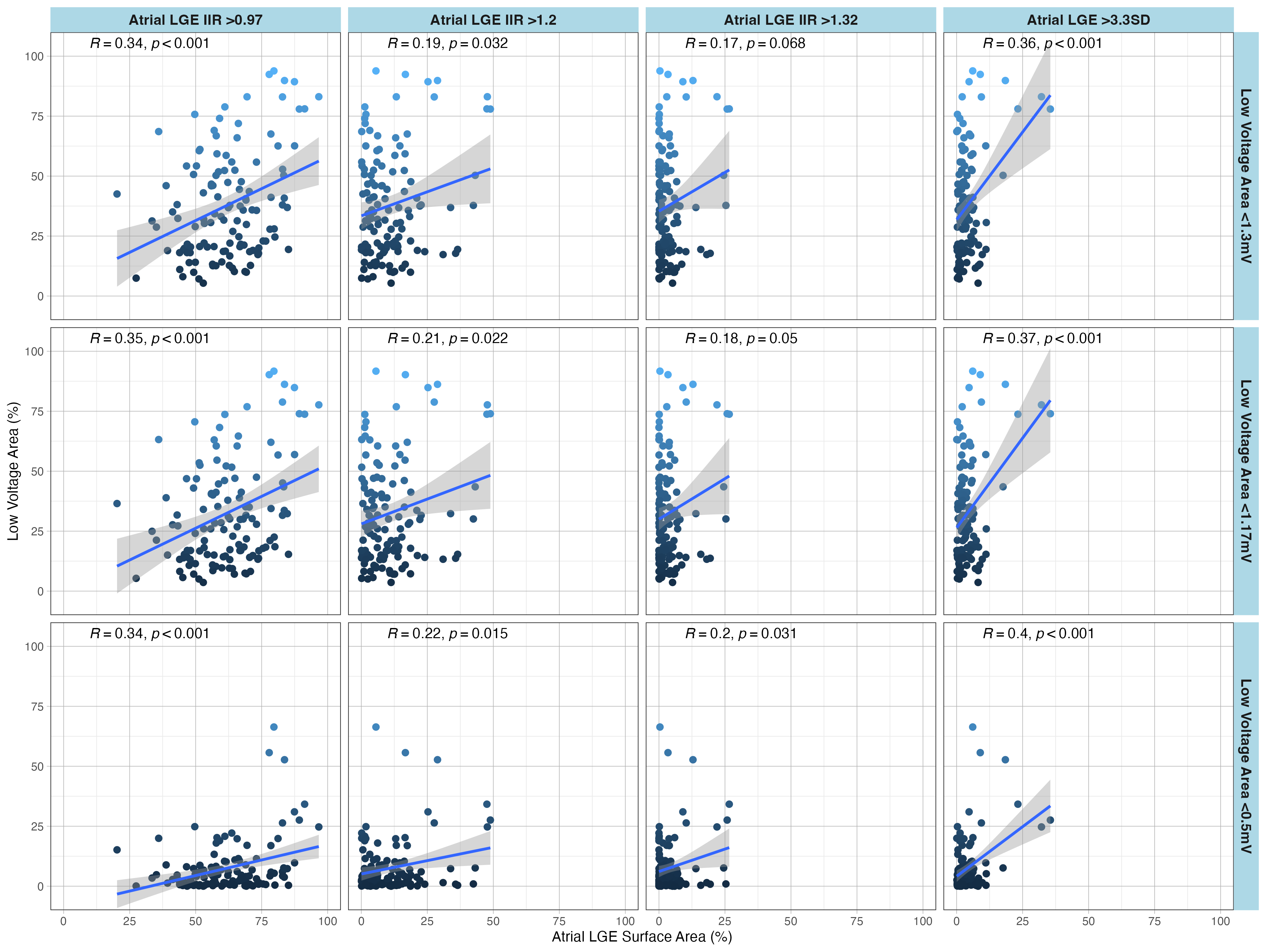


**Supplementary Figure 1:** Correlation of Atrial-EAVM and Atrial-LGE disease severity for each combination of thresholds tested. R values show Pearson’s correlation coefficient. All correlations are statistically significant. The strongest correlations exist between Atrial LGE >0.97 and each low voltage area threshold. The weakest correlation exists between Atrial LGE IIR >1.32 and each low voltage area threshold tested. LGE = late gadolinium enhancement; EAVM = electroanatomic voltage mapping; IIR = image intensity ratio.


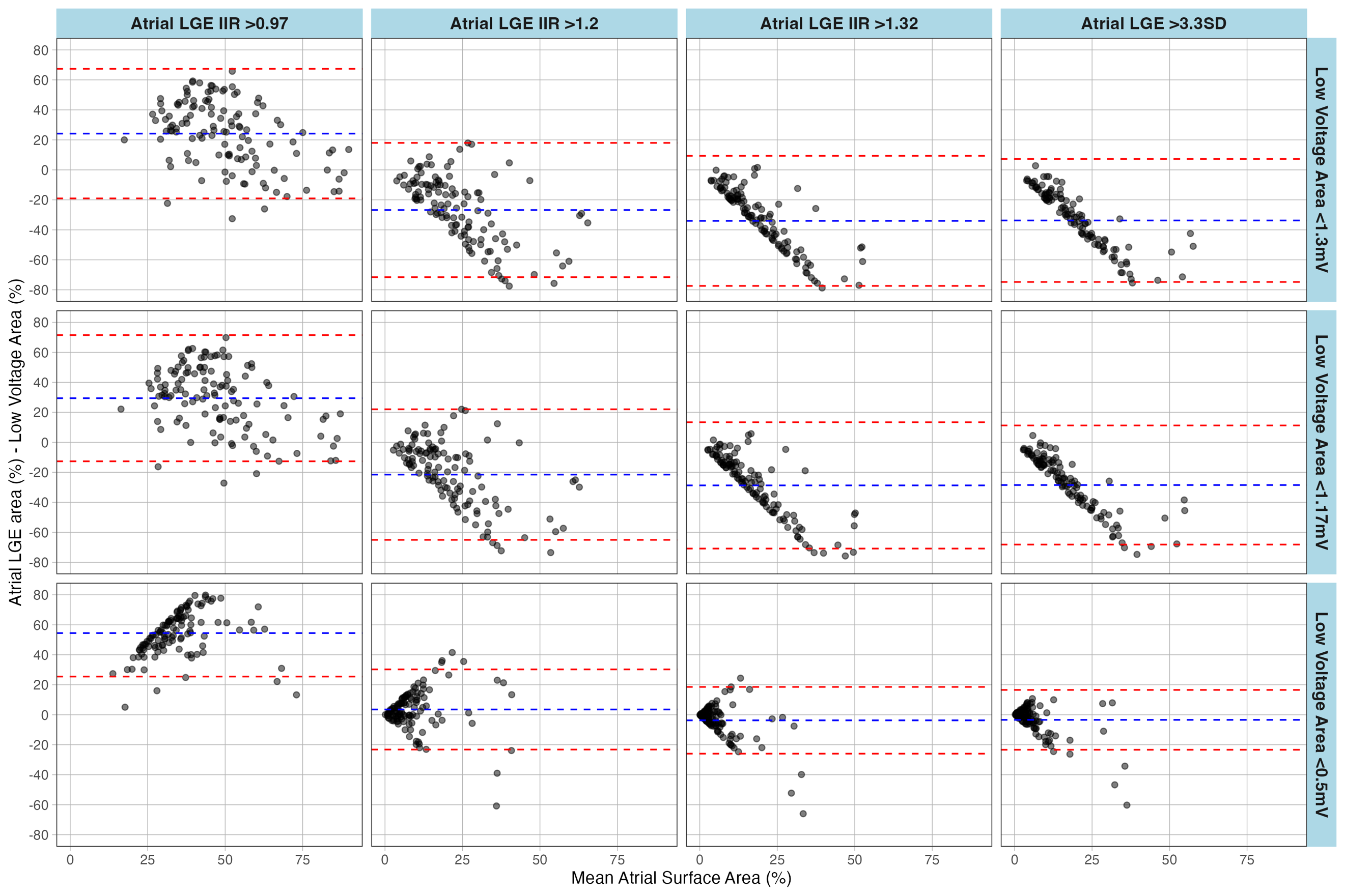


**Supplementary Figure 2:** Agreement between Atrial-EAVM and Atrial-LGE disease severity for each combination of thresholds tested. Bland Altman plots show a systematic difference between Atrial-LGE and Atrial-EAVM defined disease severity. LGE = late gadolinium enhancement; EAVM = electroanatomic voltage mapping; IIR = image intensity ratio.


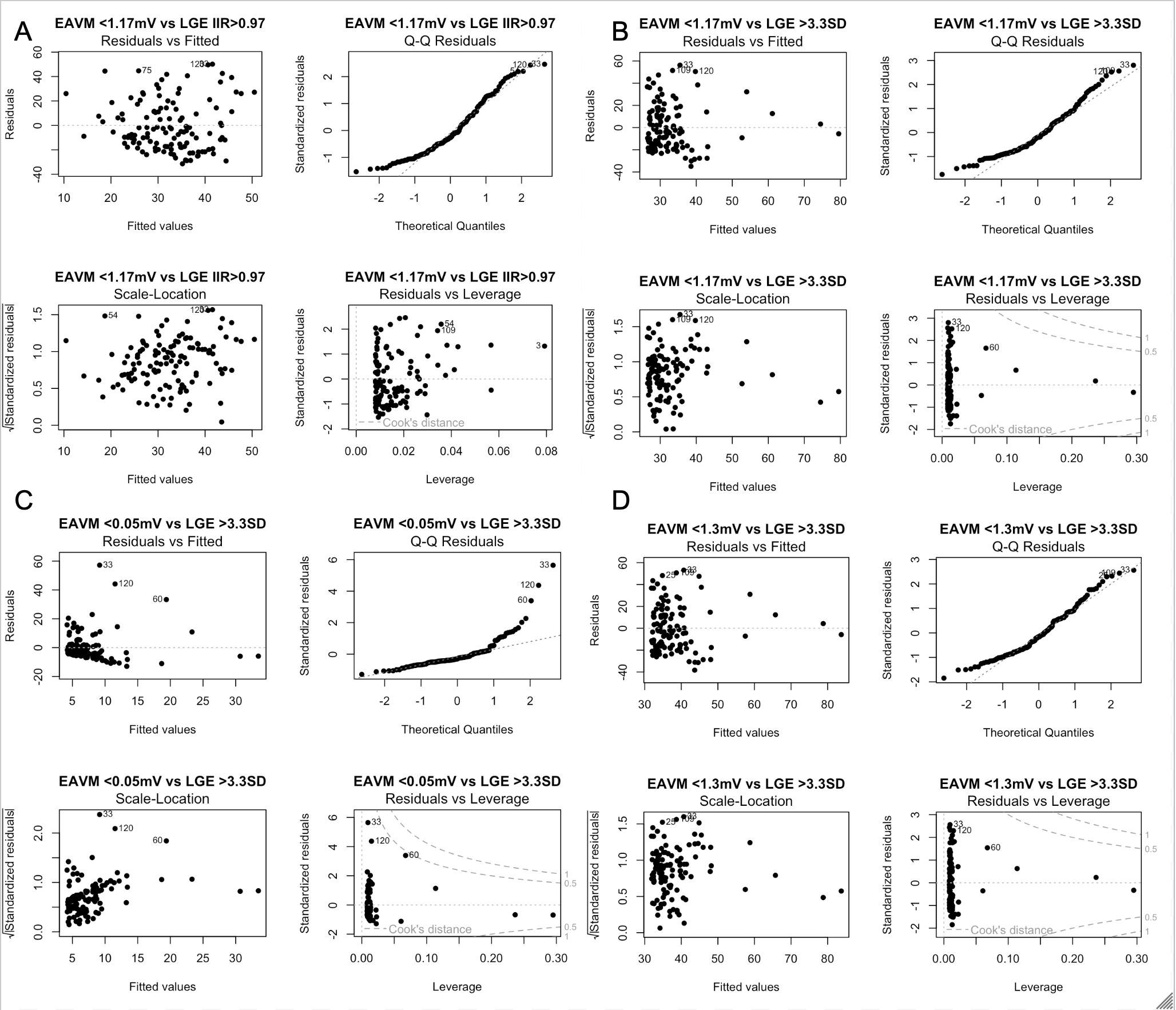


**Supplementary Figure 3:** Panels A, B, C and D show regression model statistics for relationships for between Atrial-EAVM and Atrial-LGE using 4 of the strongest Pearson correlation coefficients. For all relationships using Atrial-LGE 3.3SD (panels B, C and D), there are significant outliers contributing to the coefficient. This is not the case for panel A, showing the relationship between Atrial-EAVM LVA<1.17mV and Atrial-LGE IIR >0.97.


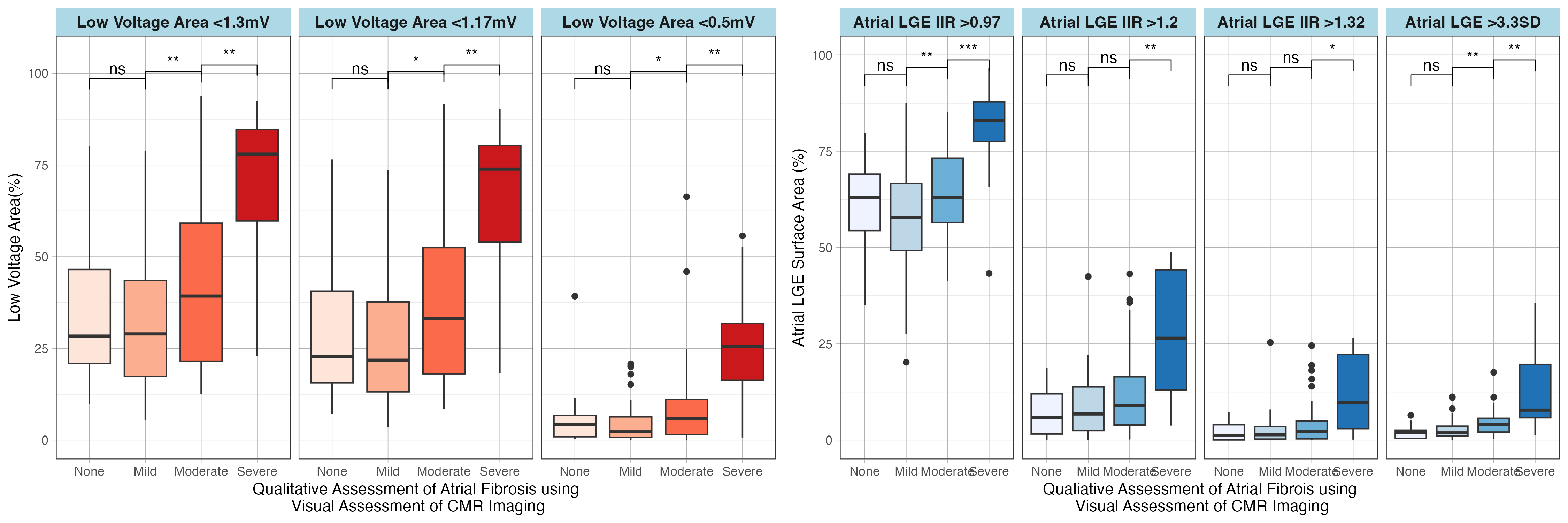


**Supplementary Figure 4:** Relationship between qualitative Atrial-LGE assessment and atrial cardiomyopathy disease severity quantification using Atrial-LGE and Atrial-EAVM. LGE = late gadolinium enhancement; EAVM = electroanatomic voltage mapping; CMR = cardiac magnetic resonance; IIR = image intensity ratio.


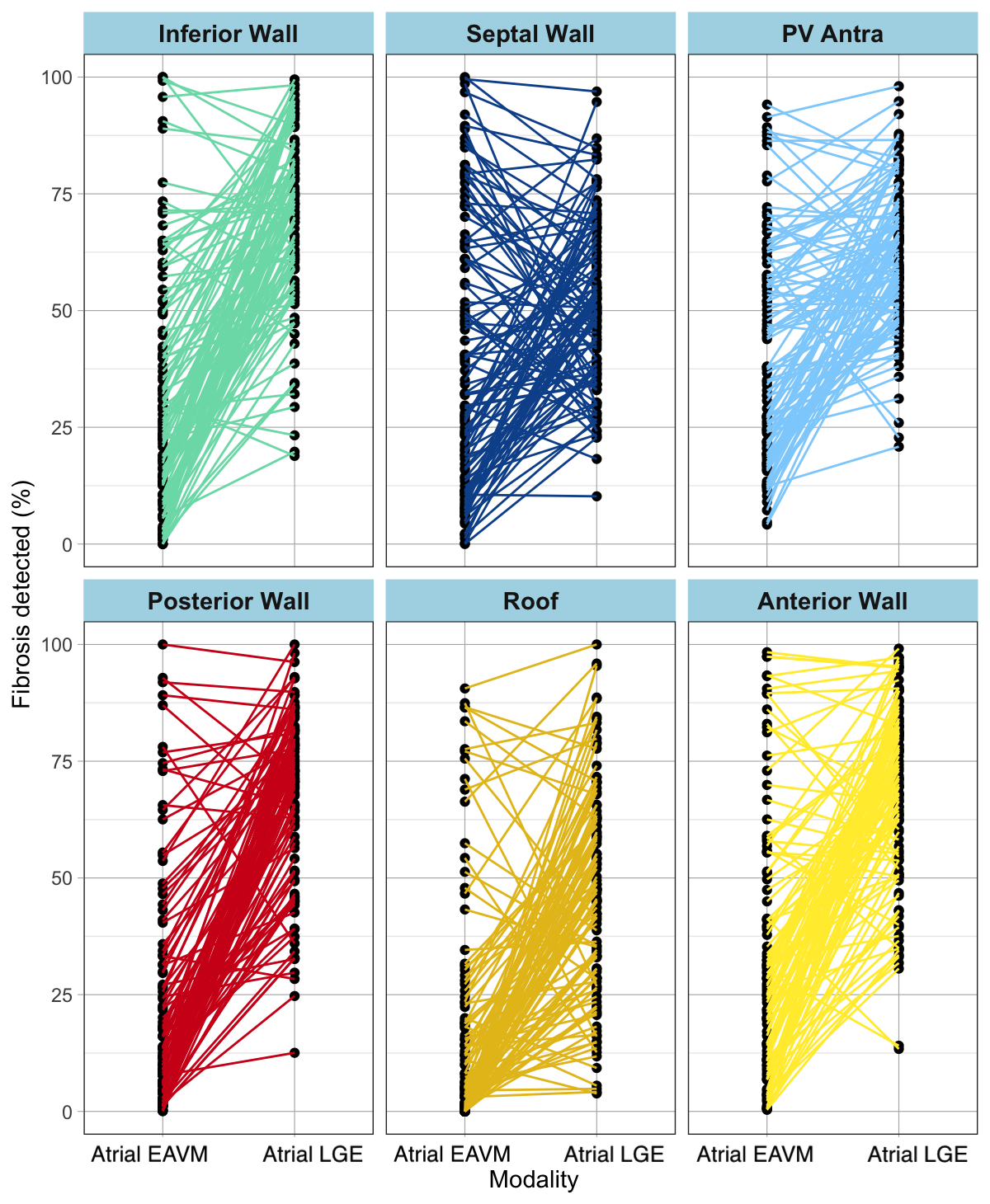


**Supplementary Figure 5:** Relationship between quantitative Atrial-LGE and Atrial-EAVM assessment per atrial region. LGE = late gadolinium enhancement; EAVM = electroanatomic voltage mapping
