# Supplementary material for: Quantification of atrial cardiomyopathy disease severity by electroanatomic voltage mapping and cardiac magnetic resonance imaging
Source: J Cardiovasc Electrophysiol. 2024 Dec 30;36(2):467–79. doi: 10.1111/jce.16462 (PMC11837893; doi:10.1111/jce.16462)
Supplement: Supplementary file 2 — Supporting information. [file JCE-36-467-s001.docx]

# Definitions of Visual Severity Category

For the visual assessment of atrial late gadolinium enhancement, four categories are used with the following definitions.

| **Atrial LGE definitions** | |
| --- | --- |
| NO fibrotic remodelling | No convincing area of atrial wall with significantly increased signal intensity. |
| MILD fibrotic remodelling | One small area of increased signal intensity.  A small area of increased signal intensity is defined as an area with a transverse extent less than half the transverse dimension of the atrial body, and present in less than half of the axial slices of the atrial transverse stack. |
| MODERATE fibrotic remodelling | More than one small area of increased signal intensity, or one large area of increased signal intensity.  A large area of increase signal intensity is defined as an area with a transverse extent greater than half the transverse width of the atrial body, or increased signal intensity present in more than half of the axial slices of the atrial transverse stack. |
| SEVERE fibrotic remodelling | More than one large area of increased signal intensity, or one large area which is contiguous and affects more than one atrial region |

# Atrial Fibrosis Visual Severity Quantification Examples

| **NO atrial fibrosis** | | | |
| --- | --- | --- | --- |
| Example 1 | Example 2 | Example 3 | Example 4 |
| 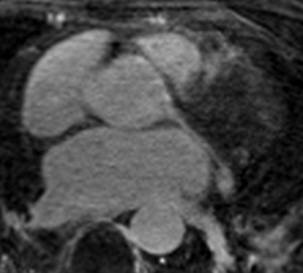 | 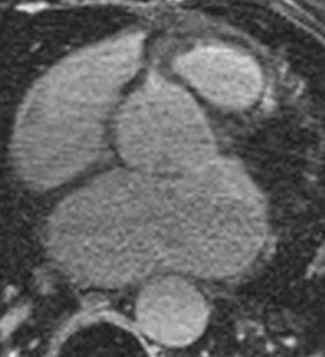 | 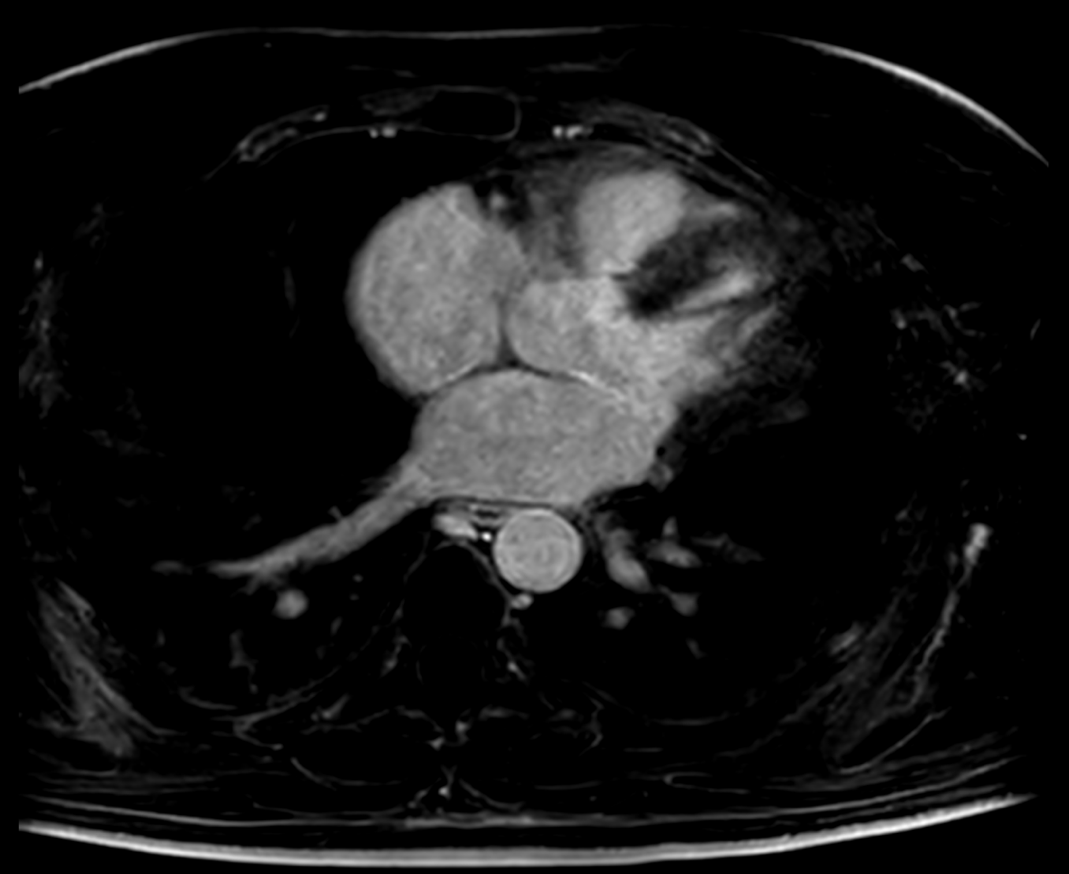 | 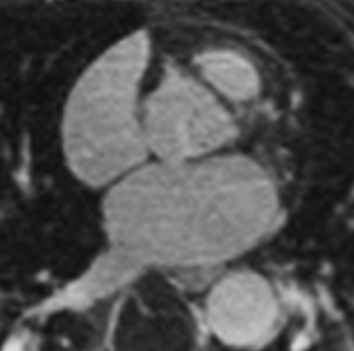 |
| **MILD atrial fibrosis** | | | |
| Example 1 | Example 2 | Example 3 | Example 4 |
| 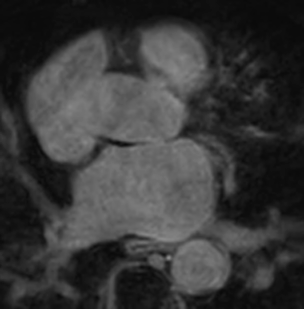 | 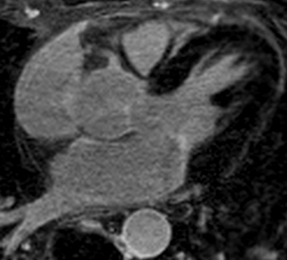 | 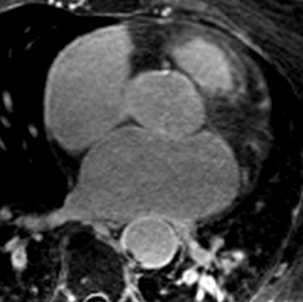 | 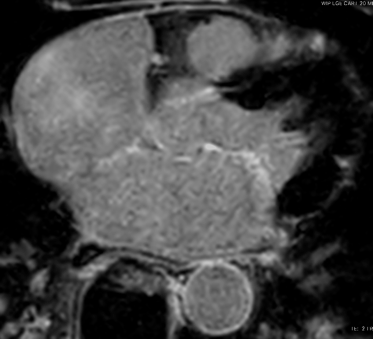 |
| **MODERATE atrial fibrosis** | | | |
| Example 1 | Example 2 | Example 3 | Example 4 |
| 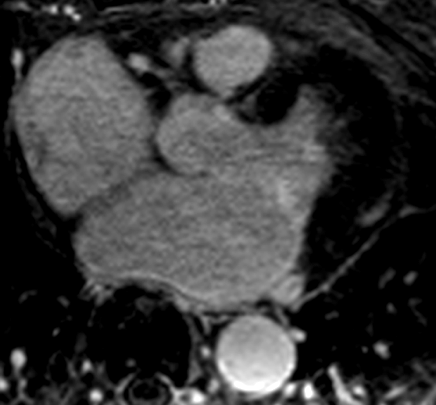 | 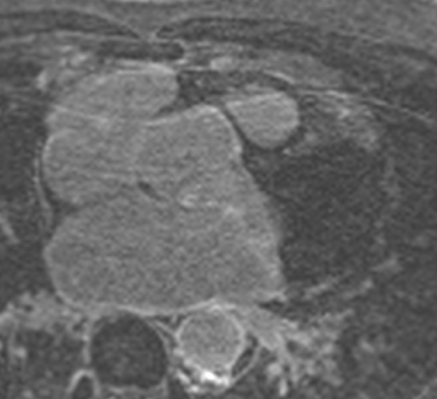 | 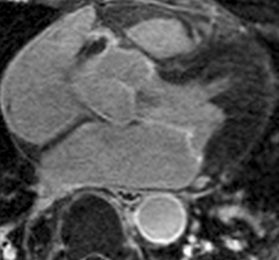 | 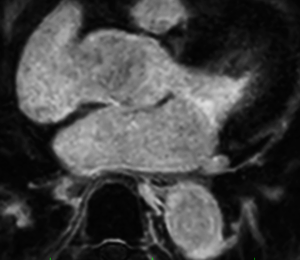 |
| **SEVERE atrial fibrosis** | | | |
| Example 1 | Example 2 | Example 3 | Example 4 |
| 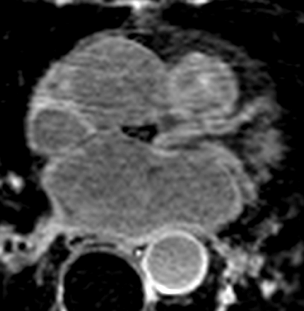 | 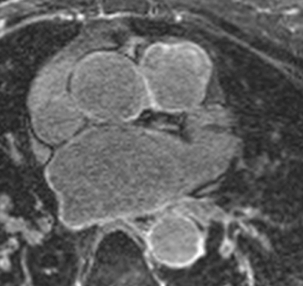 | 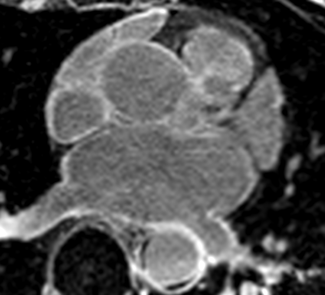 | 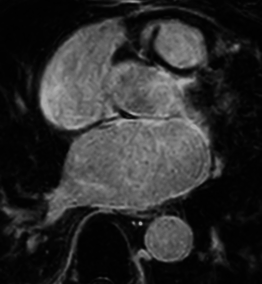 |
